# Activin A Inhibitory Peptides Suppress Fibrotic Pathways by Targeting Epithelial–Mesenchymal Transition and Fibroblast–Myofibroblast Transformation in Idiopathic Pulmonary Fibrosis

**DOI:** 10.3390/ijms26062705

**Published:** 2025-03-17

**Authors:** Victor Alexandre F. Bastos, Patrícia Tiemi Fujimura, Aline Gomes de Souza, Emília Rezende Vaz, Natieli Saito, Robinson Sabino-Silva, Luiz Ricardo Goulart, Thulio Marquez Cunha

**Affiliations:** 1 Laboratory of Experimental Biotechnology, Institute of Biotechnology, Federal University of Uberlândia, Uberlândia 38402-022, MG, Brazil; 2 Laboratory of Nanobiotechnology—Prof. Dr. Luiz Ricardo Goulart Filho, Institute of Biotechnology, Federal University of Uberlândia, Uberlândia 38402-022, MG, Brazil; tiemebr@gmail.com (P.T.F.); emiliarezendev@gmail.com (E.R.V.); natielisaito@gmail.com (N.S.); thcunha@yahoo.com.br (T.M.C.); 3 Department of Medical Imaging, Hematology, and Oncology, Ribeirão Preto Medical School, University of São Paulo, Ribeirao Preto 14040-900, SP, Brazil; alinegs@usp.br; 4 Department of Physiology, Laboratory of Nanobiotechnology—Prof. Dr. Luiz Ricardo Goulart Filho, Innovation Center in Salivary Diagnostics and Nanobiotechnology, Institute of Biomedical Sciences, Federal University of Uberlândia, Uberlândia 38402-022, MG, Brazil; robinsonsabino@gmail.com; 5 School of Medicine, Federal University of Uberlândia, Uberlândia 38408-100, MG, Brazil

**Keywords:** idiopathic pulmonary fibrosis, Activin A, synthetic peptides, epithelial–mesenchymal transition, fibroblast–myofibroblast transformation, anti-fibrotic therapy

## Abstract

Idiopathic pulmonary fibrosis (IPF) is a progressive and incurable chronic interstitial lung disease characterized by excessive fibrosis and impaired lung function. Current treatments, such as pirfenidone and nintedanib, slow disease progression but fail to halt or reverse fibrosis, highlighting the need for novel approaches. Activin A, which belongs to the TGF-β superfamily, is implicated in various fibrosis-related mechanisms, including epithelial–mesenchymal transition (EMT), a process where epithelial cells acquire mesenchymal characteristics, and fibroblast–myofibroblast transformation (FMT), in which fibroblasts differentiate into contractile myofibroblasts. It also promotes inflammatory cytokine release and extracellular matrix buildup. This study aimed to inhibit Activin A activity using synthetic peptides identified through phage display screening. Of the ten peptides isolated, A7, B9, and E10 demonstrated high binding affinity and inhibitory activity. Computational modeling confirmed that these peptides target the receptor-binding domain of Activin A, with peptide E10 exhibiting superior efficacy. Functional assays showed that E10 reduced cell migration, inhibited EMT in A549 cells, and suppressed FMT in fibroblast cultures, even under pro-fibrotic stimulation with TGF-β. These findings underscore the therapeutic potential of targeting Activin A with synthetic peptides, offering a promising avenue for IPF treatment and expanding the arsenal of anti-fibrotic strategies.

## 1. Introduction

Idiopathic pulmonary fibrosis (IPF) is a chronic, progressive interstitial lung disease characterized by the excessive deposition of extracellular matrix (ECM) in the lung, leading to irreversible architectural distortion and impaired gas exchange [1,2]. With a median survival of 3–5 years after diagnosis, and impacting more than 3 million individuals worldwide, IPF remains one of the most devastating fibrotic disorders, significantly impacting patients’ quality of life and healthcare systems worldwide [3,4]. Currently, antifibrotic drugs for IPF, mainly pirfenidone and nintedanib, which target the downstream pathways of fibrosis, are associated with significant side effects, and can only slow IPF progression, making lung transplants the only curative treatment available [5,6,7,8].

Among the molecular mediators implicated in IPF, Activin A, a member of the transforming growth factor-beta (TGF-β) superfamily, has garnered significant attention due to its dual role in tissue repair and fibrosis [9,10,11]. Activin A is a dimeric protein composed of two β subunits linked by disulfide bonds. It signals through activin receptor type IIA (ActRIIA) and type IIB (ActRIIB) in complex with type I receptors [12,13]. In healthy lungs, Activin A plays a key role in regulating immune responses, epithelial integrity, and tissue repair. However, in IPF, its dysregulation contributes to pathological processes, including epithelial–mesenchymal transition (EMT), fibroblast–myofibroblast transformation (FMT), and excessive ECM production [10,14].

The role of Activin A in IPF pathogenesis is multifaceted and critical. While it is essential for normal lung remodeling and immune responses, including epithelial regeneration and controlled fibroblast activity, its dysregulation in IPF significantly contributes to disease progression. Elevated Activin A expression is observed in both the serum and lung tissues of IPF patients and is strongly correlated with disease severity and poor prognosis [10,14]. Activin A directly drives fibroblast–myofibroblast transformation (FMT), a hallmark of IPF, by promoting myofibroblast differentiation and enhancing their resistance to apoptosis, resulting in persistent matrix deposition [15,16]. Furthermore, it amplifies fibrotic patterns by initiating a pro-inflammatory cascade, stimulating cytokines such as IL-6 and TGF-β1, which exacerbate inflammation and tissue remodeling [12]. Importantly, the natural antagonist follistatin, which normally regulates Activin A activity, is insufficient to counteract its overexpression in IPF, highlighting a critical imbalance in regulatory mechanisms [17,18]. This dysregulation establishes Activin A as a central mediator of the fibrotic process and a compelling target for therapeutic intervention.

Activin A’s centrality in IPF pathogenesis establishes it as a promising therapeutic target. The inhibition of Activin A signaling disrupts multiple fibrotic pathways, including FMT and inflammatory signaling, offering a multi-faceted approach to fibrosis attenuation [10]. Despite the promising potential of targeting Activin A, current approaches, such as follistatin-based therapies, face significant challenges, including limited stability, poor tissue penetration, and insufficient efficacy in fibrotic environments [17,19,20]. Most studies investigating Activin A inhibition have focused on monoclonal antibodies, receptor-based inhibitors, and follistatin derivatives, with limited research specifically exploring peptide-based inhibitors. These limitations highlight the need for alternative strategies that combine high specificity with robust inhibitory activity. Synthetic peptides, with their ability to precisely target key binding regions on Activin A, offer a novel and potentially more effective therapeutic solution.

Building on this rationale, this study hypothesizes that synthetic peptides designed to target Activin A can inhibit its pro-fibrotic activity. By employing phage display technology, we identified peptide candidates with high specificity for Activin A and evaluated their efficacy in disrupting FMT and ECM deposition. These findings aim to expand the therapeutic landscape for IPF and contribute to the development of targeted antifibrotic therapies.

## 2. Results

### 2.1. Peptide Selection by Phage Display

Phage clones were screened against immobilized recombinant human Activin A to identify peptides with high binding specificity and inhibitory potential. A competitive elution strategy using a neutralizing anti-Activin A antibody enriched for clones with strong binding affinity and inhibitory potential was employed over three selection rounds. A total of 46 phage clones were isolated after three rounds of selection. From these, 23 clones with an ELISA (Enzyme-Linked Immunosorbent Assay) index higher than 2 were selected for DNA sequencing (Supplementary Figure S1), which revealed 10 unique peptide sequences (Supplementary Table S1).

**Figure 1:**
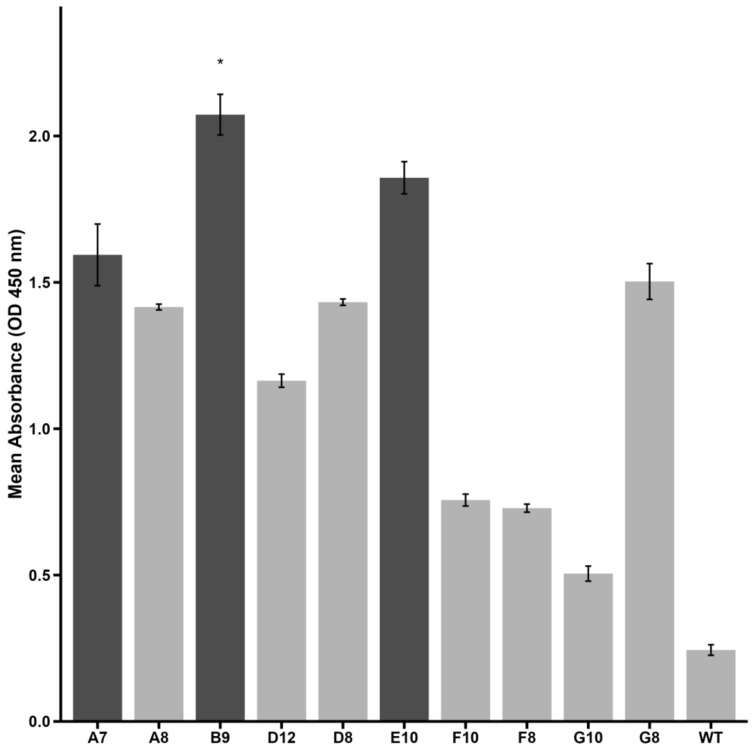
Binding affinity of selected phage clones to Activin A as measured by phage–ELISA. Optical density (OD) values at 450 nm are shown for each peptide. Clone B9 demonstrated significantly higher reactivity compared to wild-type phage (negative control) (* *p* < 0.05, *Kruskal–Wallis test with Dunn’s post hoc*). Clones A7 and E10 presented an ELISA index > 2 and are also highlighted. Darker bars represent high-binding peptides selected for further investigation. Data are presented as mean ± SE from triplicate experiments.

The binding capabilities of selected clones were assessed using phage–ELISA (Figure 1). Among the tested clones, A7, B9, and E10 exhibited significantly higher binding affinity to Activin A compared to the wild-type phage control (displaying no peptide on its pIII protein). Clone B9 exhibited the highest optical density (OD) value, indicating its superior binding capability, followed by clones E10 and A7, respectively.

These findings suggest that A7, B9, and E10 are strong candidates for further evaluation of their ability to inhibit Activin A-mediated processes. Subsequent structural and functional analyses were conducted to validate their therapeutic potential.

### 2.2. Structural Analysis and Interaction of Selected Peptides with Activin A

**Figure 2:**
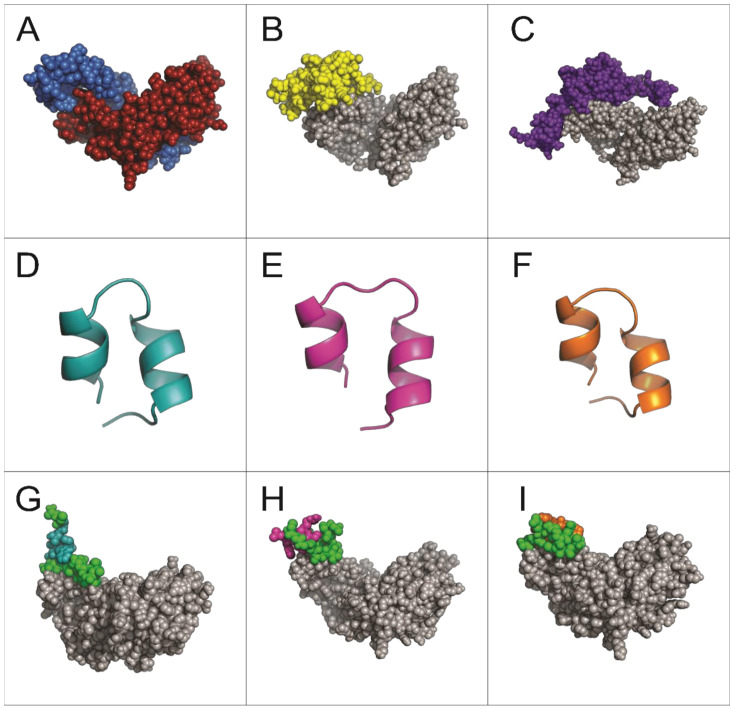
Structural predictions of molecules and interactions analyzed in this study. (**A**) Dimeric structure of Activin A (PDB: 2ARV). (**B**) Interaction of Activin A (gray) with its receptor ActRIIB (yellow). (**C**) Interaction of Activin A (gray) with follistatin (purple). (**D**–**F**) Structural predictions of peptides A7, B9, and E10, respectively. (**G**–**I**) Predicted interactions of Activin A (gray) with peptides A7, B9, and E10, respectively. The conserved region corresponding to the pIII protein sequence of M13 phage is shown in green for all peptides.

Peptides A7, B9, and E10 were synthesized using a template based on the pIII phage protein to preserve the conformation observed during the screening process. In silico docking simulations using three-dimensional models were performed to assess peptide binding mechanisms and structural interactions with Activin A (Figure 2).

The simulations revealed that all three peptides bind to the same region of the Activin A molecule (Figure 2G–I), overlapping with the binding site for the Activin A receptor ActRIIB and its natural inhibitor follistatin (Figure 2B,C). This shared binding region highlights the potential of these peptides to competitively inhibit Activin A signaling.

**Figure 3:**
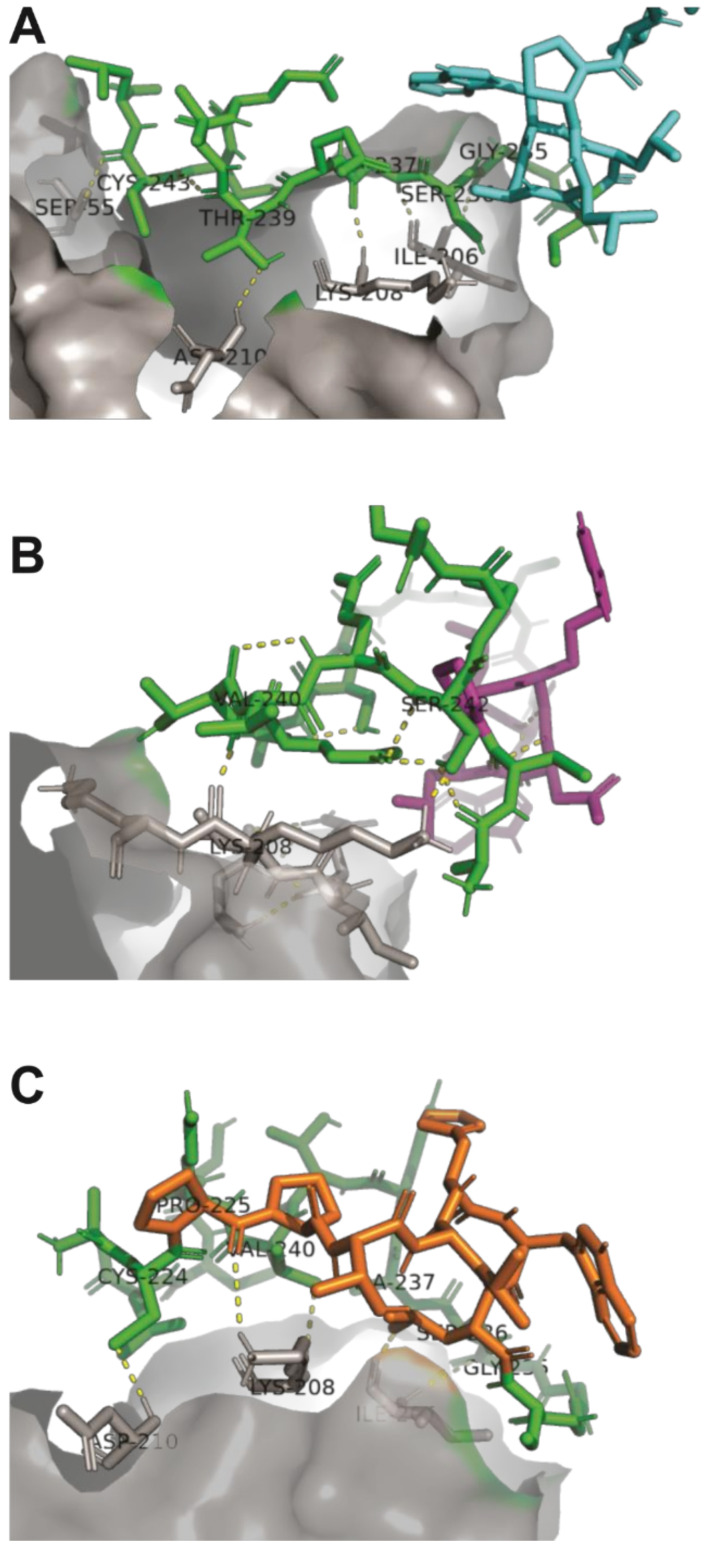
Binding interactions between peptides A7, B9, and E10 and the Activin A molecule. (**A**) The pIII protein region of peptide A7 interacts with isoleucine (I206), lysine (K208), and aspartic acid (D210) within the ActRIIB recognition site of Activin A. (**B**) Peptide B9 forms a covalent interaction exclusively with lysine (K208) in the ActRIIB recognition site. (**C**) Peptide E10 interacts with isoleucine (I206), lysine (K208), and aspartic acid (D210) in the ActRIIB recognition site, with a specific contribution from proline in its sequence, enhancing its binding stability. The conserved pIII region is shown in green, Activin A in gray, and the unique peptide regions in cyan (A7), purple (B9), and orange (E10).

Unlike peptides A7 and B9, which predominantly interact through the conserved pIII sequence, peptide E10 exhibited a distinct interaction involving one specific amino acid from its unique sequence. This unique residue contributes to the peptide’s enhanced binding affinity and specificity by forming stable interactions with critical residues on Activin A, isoleucine (I206), lysine (K208), and aspartic acid (D210) (Figure 3). This enhanced binding specificity suggests that E10 may exhibit superior inhibitory potential against Activin A-mediated processes.

These findings provide insights into the structural basis of peptide–Activin A interactions and highlight peptide E10 as the most promising candidate for further functional evaluation.

### 2.3. Cytotoxicity of Selected Peptides

**Figure 4:**
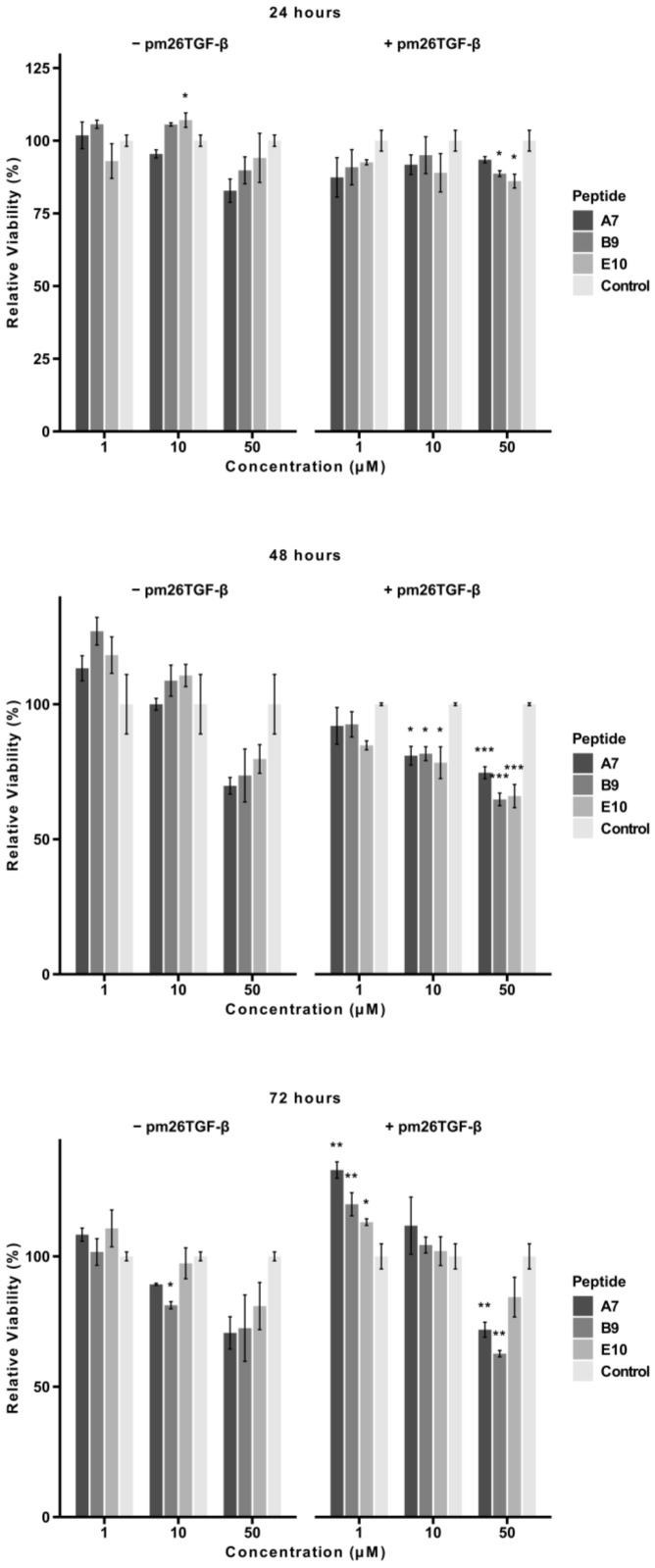
Cytotoxic effects of peptides A7, B9, and E10 on A549 cells. Cells were treated with peptides at concentrations of 1 µM, 10 µM, and 50 µM in the presence or absence of pm26TGF-β1 (1 ng/mL) as positive and negative controls, respectively. Cell viability was monitored at 24, 48, and 72 h. Significant cytotoxicity was observed for all peptides at 50 µM at 48 h. After 72 h, only peptides A7 and B9 presented significant cytotoxicity at 50 µM in the presence of pm26TGF-β1. Statistical analysis was conducted using pairwise *t*-tests with *p*-values adjusted by the False Discovery Rate (FDR) method (* adjusted *p* < 0.05, ** adjusted *p* < 0.01, *** adjusted *p* < 0.001). Data are presented as mean ± SD from triplicate experiments.

To evaluate the potential cytotoxic effects of peptides A7, B9, and E10, an MTT assay was conducted using A549 cells treated with 1 µM, 10 µM, and 50 µM of each peptide in the presence or absence of pm26TGF-β1 (1 ng/mL). The cells were monitored at 24, 48, and 72 h (Figure 4). 

After 24 h, peptides B9 and E10 exhibited significant cytotoxicity at 50 µM in the presence of pm26TGF-β1 (adjusted *p* < 0.05). In the absence of pm26TGF-β1, A7 and B9 showed more pronounced cytotoxic effects compared to E10. At lower concentrations (1 µM and 10 µM), no significant cytotoxicity was observed for any peptide. Notably, at this time point, mean cell viability for all peptides remained above 70% under all conditions (Supplementary Table S2).

At 48 h, a dose-dependent reduction in cell viability was evident. In the presence of pm26TGF-β1, particularly at 10 µM (adjusted *p* < 0.05) and 50 µM (adjusted *p* < 0.01), all peptides showed significant reductions in viability, suggesting a potential antagonistic effect between the peptides and pm26TGF-β1stimuli. In the absence of pm26TGF-β1, cytotoxic effects were observed primarily at 50 µM, with A7 and B9 showing the most pronounced reductions. Only A7 (50 µM, without pm26TGF-β1), B9, and E10 (50 µM, with pm26TGF-β1) exhibited mean cell viability below 70% at this time point (Supplementary Table S2).

After 72 h, peptides A7 and B9 demonstrated sustained cytotoxicity at 50 µM (adjusted *p* < 0.01) in the presence of pm26TGF-β1, while E10 showed no significant cytotoxic effects under the same conditions. B9 also exhibited mild cytotoxicity at 10 µM (adjusted *p* < 0.05) without pm26TGF-β1 stimulation. At this time point, only B9 (50 µM, with pm26TGF-β1) reduced mean viability below 70% (Supplementary Table S2).

Interestingly, at 1 µM and 10 µM, cell viability increased from 48 to 72 h, suggesting a potential adaptive cellular response. This effect may be associated with the activation of repair pathways and metabolic adjustments in response to initial peptide-induced stress [21]. Additionally, a decrease in extracellular peptide concentration due to degradation or internalization over time could contribute to reduced cytotoxicity [22,23]. Future studies evaluating oxidative stress markers and peptide stability in the culture medium may help clarify the mechanisms underlying this phenomenon.

These findings highlight the dose- and time-dependent cytotoxic profiles of peptides A7 and B9, particularly at higher concentrations and in the presence of pm26TGF-β1. In contrast, E10 maintained minimal cytotoxicity across most conditions, reinforcing its potential as a safer therapeutic candidate.

### 2.4. Cellular Migration

**Figure 5:**
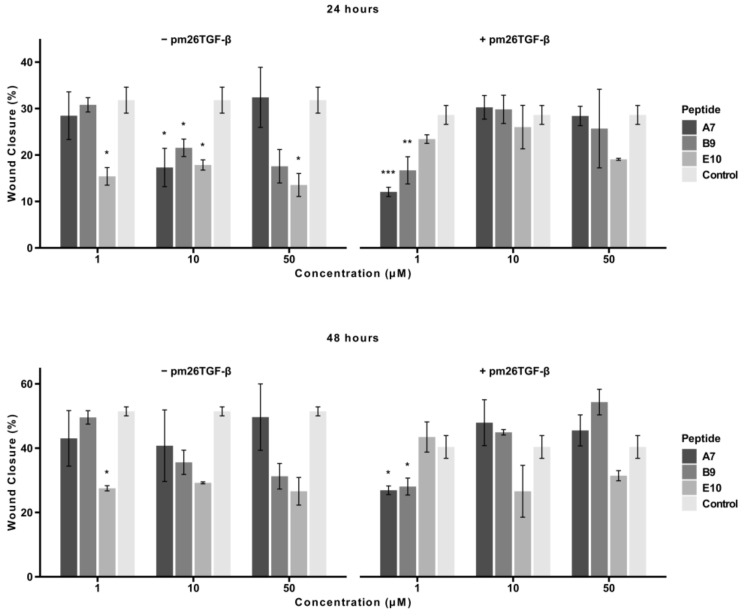
Effects of peptides A7, B9, and E10 on cellular migration. Scratch assays were performed using A549 cells treated with peptides at 1 µM, 10 µM, and 50 µM, in the presence or absence of pm26TGF-β1 (1 ng/mL). Control cells were treated with DMEM medium only in the − pm26TGF-β group and DMEM medium supplemented with pm26TGF-β1 (1 ng/mL) in the + pm26TGF-β1 group. Wound closure was monitored at 24 and 48 h. Peptide E10 exhibited the most consistent and significant inhibitory effects on cellular migration across conditions, particularly at moderate concentrations and in the presence or absence of pm26TGF-β1. Statistical analysis was conducted using pairwise *t*-tests with *p*-values adjusted by the False Discovery Rate (FDR) method (* adjusted *p* < 0.05, ** adjusted *p* < 0.01, *** adjusted *p* < 0.001). Data are presented as mean ± SD from triplicate experiments.

The effects of peptides A7, B9, and E10 on cell migration were assessed using a scratch assay with A549 cells. Cells were treated with peptides at concentrations of 1 µM, 10 µM, and 50 µM, in the presence or absence of pm26TGF-β1 (1 ng/mL). Images of the wound area were captured at 0, 24, and 48 h, and the percentage of wound closure was quantified (Figure 5).

At 24 h, in the absence of pm26TGF-β1, E10 demonstrated a consistent and statistically significant inhibitory effect across all tested concentrations (adjusted *p* < 0.05). A7 and B9 exhibited significant inhibition only at 10 µM (adjusted *p* < 0.05). In the presence of pm26TGF-β1, A7 and B9 were most effective at 1 µM, with A7 showing stronger inhibition (adjusted *p* < 0.001) compared to B9 (adjusted *p* < 0.01). At higher concentrations (10 µM and 50 µM), E10 demonstrated the strongest inhibitory effect among the peptides.

At 48 h, E10 maintained a consistent and potent inhibitory effect at all tested concentrations in the absence of pm26TGF-β1. In contrast, in the presence of pm26TGF-β1, A7 and B9 exhibited significant inhibition only at 1 µM (adjusted *p* < 0.05), while E10 displayed the opposite trend, with greater inhibitory activity observed at 10 µM and 50 µM concentrations. This suggests a dose-dependent relationship for E10 in the presence of pm26TGF-β1, as well as a potential mechanism independent of the TGF-β signaling pathways.

Overall, peptide E10 demonstrated a more consistent and pronounced inhibitory effect on cellular migration across all conditions, particularly at moderate, non-cytotoxic concentrations. These findings underscore E10’s superior ability to inhibit cell migration and reinforce its potential as a therapeutic candidate for IPF.

### 2.5. EMT and FMT Assay

**Figure 6:**
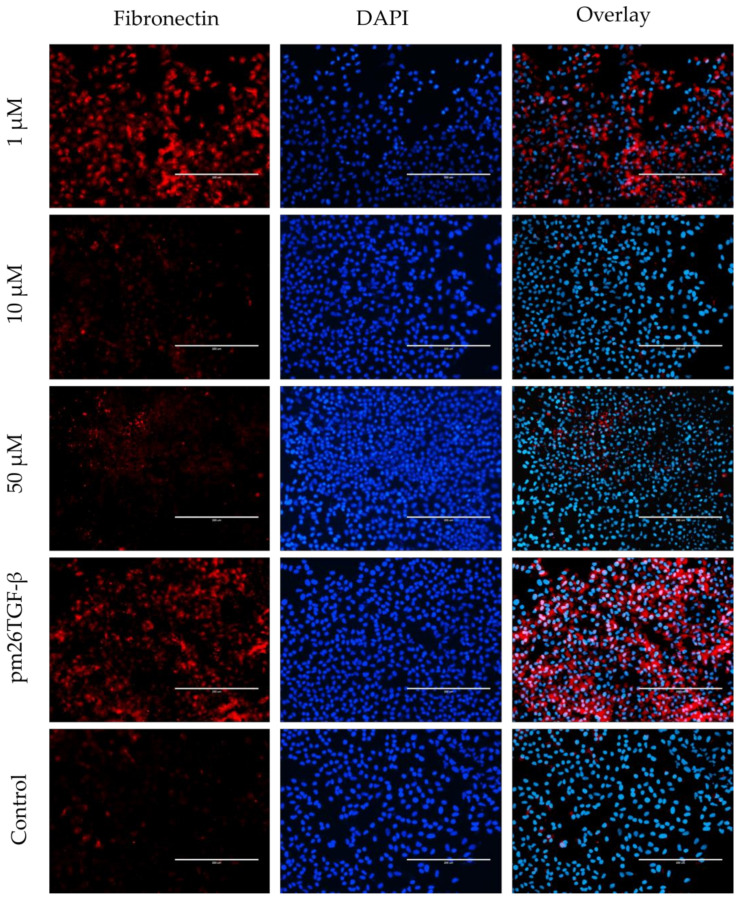
Effects of peptide E10 on epithelial–mesenchymal transition (EMT) in A549 cells. Cells were treated with E10 at 1 µM, 10 µM, and 50 µM in the presence or absence of pm26TGF-β1 (1 ng/mL), as positive and negative controls, respectively. EMT inhibition was evaluated by measuring fibronectin expression. The strongest inhibitory effects were observed at 10 µM, with reduced fibronectin levels compared to cells treated only with pm26TGF-β1. DAPI (4′,6-Diamidino-2-phenylindole) was used for nucleus visualization. Scale bar: 200 µm.

**Figure 7:**
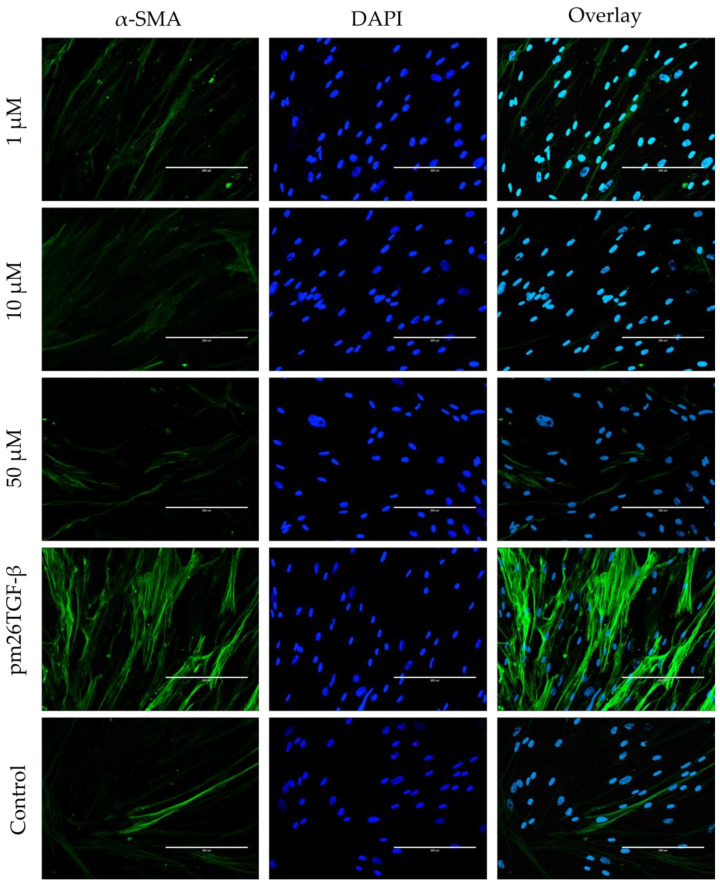
Effects of peptide E10 on fibroblast–myofibroblast transformation (FMT) in primary fibroblasts. Cells were treated with E10 at 1 µM, 10 µM, and 50 µM in the presence or absence of pm26TGF-β1 (1 ng/mL), as positive and negative controls, respectively. FMT inhibition was assessed by measuring α-smooth muscle actin (α-SMA) fiber expression. Significant inhibition of FMT was observed at 10 µM and 50 µM, with reduced α-SMA levels compared to cells treated only with pm26TGF-β1. DAPI (4′,6-Diamidino-2-phenylindole) was used for nucleus visualization. Scale bar: 200 µm.

To assess the effects of peptide E10 on epithelial–mesenchymal transition (EMT) (Figure 6) and fibroblast–myofibroblast transformation (FMT) (Figure 7), experiments were conducted using A549 cells and primary fibroblasts, respectively. Both cell types were treated with E10 at concentrations of 1 µM, 10 µM, and 50 µM, in the presence or absence of pm26TGF-β1 (1 ng/mL).

In A549 cells, E10 treatment significantly inhibited EMT, as evidenced by reduced fibronectin expression compared to cells treated only with pm26TGF-β1 (Figure 6). The inhibitory effect was most pronounced at 10 µM, aligning with previous observations of E10’s activity in cytotoxicity and migration assays. At 50 µM, E10 also reduced fibronectin levels, although cytotoxic effects at this concentration may partially contribute to this observation. Conversely, at 1 µM, the inhibitory effect was less consistent, indicating a dose-dependent response for E10 in mitigating EMT.

In primary fibroblasts, E10 effectively inhibited FMT, as shown by the decreased expression of α-smooth muscle actin (α-SMA) fibers compared to cells treated only with pm26TGF-β1 (Figure 7). The inhibitory effects were most prominent at 10 µM and 50 µM, with α-SMA levels substantially reduced at these concentrations. Interestingly, at 1 µM, E10 showed modest effects on FMT, further supporting the notion of a concentration-dependent mechanism.

Overall, E10 demonstrated robust inhibitory activity against the EMT and FMT processes at non-cytotoxic concentrations. These findings highlight its potential as a therapeutic agent to mitigate pro-fibrotic pathways in conditions like IPF, where these processes play a central role in disease progression.

## 3. Discussion

In this study, we demonstrated that peptide E10 effectively binds to Activin A, inhibiting its interaction with ActRIIB, and mitigating key fibrotic processes such as EMT, FMT, and cellular migration in vitro. Among the peptides identified through phage display, E10 exhibited high binding affinity and specificity, highlighting its potential as a therapeutic candidate for IPF.

IPF remains a life-threatening interstitial lung disease with limited therapeutic options [24,25]. The current treatments, pirfenidone and nintedanib, can only slow disease progression and have significant side effects, leaving a critical need for novel, targeted, and more efficient therapies [26,27]. The quest for more effective and better-tolerated treatments has led to the exploration of novel therapeutic targets. Emerging therapies aim to address the complex pathogenesis of IPF by targeting the specific molecular pathways involved in fibrosis. These include agents that inhibit fibrogenic mediators and modulate immune responses, as well as novel drug delivery systems, offering hope for improved patient outcomes [24,28,29,30,31].

Under physiological conditions, Activin A regulates immune responses, epithelial integrity, and wound healing, contributing to normal lung remodeling after injury [9,11,32,33]. However, in fibrotic diseases such as IPF, its dysregulation drives pathological processes, including EMT, FMT, and excessive ECM deposition [19,34,35,36]. Elevated Activin A levels in the serum and lung tissue of IPF patients highlight its role in fibrosis progression [10,14]. While natural inhibitors like follistatin can neutralize Activin A, their limited efficacy in fibrotic environments necessitates alternative approaches [17,19,37,38]. Receptor-based inhibitors show promise in preclinical models but face challenges in maintaining therapeutic specificity due to Activin A’s involvement in diverse biological processes [39,40,41,42,43].

Therapeutic peptides, such as those identified in this study, hold significant potential as antifibrotic agents. Compared to small molecules or monoclonal antibodies, peptides often exhibit favorable safety profiles, reduced immunogenicity, and the ability to modulate protein–protein interactions with high specificity [44,45]. Furthermore, their relatively small size and structural flexibility enhance their ability to bind complex or shallow target interfaces, such as the receptor-binding domains of Activin A. These properties make therapeutic peptides particularly promising for diseases like IPF, where the targeted inhibition of pathological pathways must be achieved without broadly impairing essential physiological functions [46].

Binding simulations further revealed that our peptides occupy the Activin A binding region, and are thus critical for interactions with its receptor ActRIIB and its natural inhibitor, follistatin (Figure 2 and Figure 3), suggesting their potential in disrupting pathological signaling pathways effectively in IPF [20,47,48]. Structural analysis and binding simulations revealed E10 potential as a therapeutic agent. Unlike A7 and B9, which showed moderate interaction strength, E10 formed stable and specific contacts with critical residues on Activin A, including lysine (K102), aspartic acid (D104), and isoleucine (I100), all of which correspond to the receptor recognition site of ActRIIB, as demonstrated by Mason et al. [47]. These interactions also involve five amino acids of E10’s structure, notably including proline, which is exclusive to its specific sequence. This unique binding profile highlights E10’s superior specificity compared to A7 and B9, whose interactions occurred exclusively through residues common to the pIII region of the M13 phage. Although A7 interacted with lysine (K102), aspartic acid (D104), and isoleucine (I100) in the ActRIIB binding site, these interactions were mediated entirely by the non-specific pIII region, which is shared across all of the selected peptides. Interestingly, A7 showed the highest frequency (43.48%) among the 10 selected sequences, suggesting that its final structural conformation played a key role during the selection process, despite its lack of unique target-binding regions. Similarly, B9 exhibited covalent interaction only with lysine (K102), but again, this was confined to the pIII region. By contrast, E10 demonstrated a more robust and unique interaction profile, engaging both shared and specific residues in the ActRIIB binding site. This specificity is critical, as it minimizes off-target effects, a common limitation of broader antifibrotic drugs. By targeting the same binding region used by follistatin and ActRIIB, E10 could act as a competitive inhibitor, providing a novel mechanism for selectively modulating Activin A activity in fibrotic environments [17,20,49,50].

In the presence of TGF-β, increased cytotoxicity was observed for all peptides, particularly at higher concentrations and longer exposure times. This suggests that TGF-β may sensitize cells to peptide-induced cytotoxicity. Importantly, the enhanced cytotoxicity observed under these conditions could reflect the peptides’ ability to interfere with TGF-β-driven signaling pathways. TGF-β is known to regulate various cellular processes, including promoting EMT and cell proliferation in certain contexts [51]. The observed cytotoxicity profiles may, therefore, indicate the peptides’ dual capacity to inhibit pathological signaling pathways while selectively targeting proliferative responses induced by TGF-β1. While all three tested peptides have the ability to inhibit Activin A, E10 demonstrated a superior profile. Activin A, which shares downstream pathways with TGF-β, plays a central role in fibrosis by promoting EMT, FMT, and extracellular matrix deposition. By targeting the shared receptor-binding site of Activin A and potentially modulating TGF-β-related pathways, E10 demonstrated a capacity to disrupt fibrosis-promoting mechanisms while maintaining low cytotoxicity under non-stimulated conditions. Additionally, beyond Activin A and TGF-β1, other pro-inflammatory and pro-fibrotic mediators contribute to IPF. IL-6 and TNF-α have been reported to amplify fibrotic signaling through their interaction with TGF-β1 [52,53,54]. Furthermore, progranulin has been found to be elevated in IPF patients, suggesting its role in modulating the inflammatory microenvironment and exacerbating fibrosis [14]. These mediators highlight the complex network involved in fibrosis progression and reinforce the significance of targeting Activin A as a key regulatory factor.

The timeframe of TGF-β’s action in A549 cells is particularly relevant to these findings. Studies suggest that TGF-β1 can induce EMT within 24 h, with more pronounced effects over extended periods [37,55,56]. This aligns with our observation of increased cytotoxicity at 48 and 72 h, potentially reflecting a cumulative disruption of TGF-β-mediated signaling by the peptides. Furthermore, the known release of Activin A by A549 cells under inflammatory conditions adds another layer of complexity, as the peptides’ inhibitory effects on Activin A could further influence cell survival and proliferation [10,57]. Taken together, these findings highlight the intricate interplay between TGF-β signaling, Activin A inhibition, and peptide cytotoxicity. E10’s ability to mitigate pro-fibrotic processes with minimal off-target effects positions it as a promising therapeutic candidate for fibrotic diseases like IPF.

Cellular migration is a critical process in fibrosis progression, facilitating myofibroblast recruitment and ECM deposition [58,59]. In this study, peptide E10 demonstrated the most consistent and pronounced inhibitory effects on cellular migration across most conditions, reinforcing its potential as a therapeutic candidate for IPF. Notably, E10 exhibited significant inhibition at moderate, non-cytotoxic concentrations, a key advantage for therapeutic applications requiring long-term safety. At 24 h, E10 effectively inhibited migration in both the presence and absence of pm26TGF-β1, with its strongest effects observed at 10 µM and 50 µM concentrations. Interestingly, A7 and B9 were more effective at 1 µM in the presence of pm26TGF-β1 and demonstrated an antagonistic effect at higher concentrations. Similar results were observed by Horan et al., serving to highlight the importance of a therapeutic window [60].

EMT is a dynamic cellular process associated with increased motility and invasiveness. In IPF, EMT contributes to the transformation of type II alveolar epithelial cells and the creation of a pro-fibrotic microenvironment that promotes fibroblast activation and differentiation into myofibroblasts [31,61,62]. This process can be evaluated by the expression of extracellular fibronectin. Our results demonstrated that E10, at concentrations of 10 µM and 50 µM, effectively inhibited extracellular fibronectin expression in A549 cells, even following stimulation with pm26TGF-β1 (Figure 6).

FMT is crucial in IPF development, as the inflammatory imbalance creates a microenvironment conducive to the continuous recruitment of fibroblasts and their differentiation into myofibroblasts, which are primarily responsible for ECM deposition leading to scar tissue formation [16,58,63]. Here, we demonstrated that E10 significantly reduced α-SMA expression in primary fibroblasts from healthy donors after pm26TGF-β1 treatment (Figure 7).

EMT and FMT are pivotal drivers of fibrosis, converting epithelial and fibroblast cells into ECM-secreting myofibroblasts [64,65]. Activin A has been implicated in promoting both EMT and FMT, contributing to fibrosis progression, with elevated levels of Activin A observed in various fibrotic diseases [10,15,66,67]. The ability of E10 to target multiple fibrotic mechanisms, including EMT, FMT, and cellular migration, through Activin A inhibition makes it an attractive candidate for IPF therapy. Current antifibrotic drugs primarily target downstream pathways or broad signaling cascades, often resulting in limited efficacy and adverse effects [8,24,30,68]. In contrast, E10’s specificity for Activin A offers a more focused approach, potentially reducing off-target effects while addressing critical molecular events that drive fibrosis progression.

Despite these promising findings, some questions remain. First, while in vitro studies provide valuable insights, the therapeutic potential of E10 must be validated in vivo. Animal models of pulmonary fibrosis, such as bleomycin-induced models, could assess the peptide’s ability to reduce ECM deposition, improve lung architecture, and enhance respiratory function. Additionally, the pharmacokinetics and stability of E10 in systemic circulation should be evaluated to determine its suitability for clinical use. Second, the potential synergy between E10 and existing antifibrotic therapies, such as nintedanib and pirfenidone, also warrants exploration, as combination treatments could offer enhanced efficacy. Third, it is important to identify or develop safe and efficient mechanisms for direct delivery of the peptide to the fibrotic lung. Targeted delivery systems, such as nanoparticle-based carriers or inhalable formulations, could enhance E10’s therapeutic efficacy by concentrating its action at the site of fibrosis while minimizing off-target effects. These strategies would not only improve the peptide’s bioavailability, but also mitigate potential systemic side effects, ensuring its clinical applicability in the long-term management of IPF.

Finally, this study highlights the utility of phage display technology in identifying novel therapeutic candidates. The success of E10 in targeting Activin A demonstrates the potential of this approach for developing peptide inhibitors against fibrotic pathways. By mitigating EMT, FMT, and cellular migration, E10 offers a promising antifibrotic strategy. Future studies should focus on in vivo validation and clinical translation to further explore its therapeutic potential.

## 4. Materials and Methods

### 4.1. Reagents and Materials

The M13 phage library and *Escherichia coli* ER2738 were sourced from the PhD-7mer Phage Display Peptide Library Kit (New England Biolabs Inc., Ipswich, MA, USA). Recombinant human Activin A (Cat# 592006) and anti-Activin A antibody (Cat# 693603) were purchased from BioLegend (San Diego, CA, USA).

#### Cell Lines and Primary Culture

For cell culture assays, A549 cells (ATCC^®^ CCL-185™) and human primary fibroblasts were used. Primary fibroblasts were isolated from lung bronchoscopy samples collected from healthy donors who underwent bronchoscopy for non-IPF-related conditions at the University Hospital of the Federal University of Uberlândia (Uberlândia—Brazil).

The Ethics Committee on Human Research at the Federal University of Uberlândia (CEP 0932/2015) approved all procedures. Written informed consent was obtained from all participants prior to sample collection, ensuring adherence to ethical standards.

For pro-inflammatory and pro-fibrotic stimuli in cell cultures, we used pm26TGF-β1, a synthetic peptide developed by our group that mimics the effect of TGF-β1 [69].

### 4.2. Phage Display Screening

The phage display screening process followed the procedures described by Barbas et al. [70]. To select high-affinity binding peptides to Activin A, we used a PhD-7mer Phage Display Peptide Library Kit (New England Biolabs Inc., Ipswich, MA, USA). A high-binding 96-well plate (Greiner Bio-One, Kremsmünster, Austria) was coated with 100 µL of recombinant human Activin A (10 µg/mL) diluted in 0.1 M NaHCO₃ buffer (pH 8.6) and incubated overnight at 4 °C. Excess and weakly bound Activin A molecules were removed by two washes with PBS 1X (137 mM NaCl, 10 mM phosphate, 2.7 mM KCl, pH 7.4). Blocking was performed with 300 µL/well of 5% (*w*/*v*) BSA in PBS 1X for 1 h at room temperature (RT) with agitation (200 rpm). After blocking, wells were washed six times with 200 µL of PBS-T 0.05% (PBS supplemented with 0.05% Tween-20, St. Louis, MO, USA). A total of 10 µL of the phage library was diluted in 90 µL of PBS-T 0.05% and added to the Activin A-coated well. The plate was incubated for 1 h at RT with agitation (200 rpm). Unbound phages were removed by washing the well 10 times with 300 µL of PBS-T 0.05%.

To recover specific phages, bound phages were competitively eluted using 100 µL of anti-Activin A neutralizing antibody (0.1 µg/µL) diluted in PBS 1X. Elution was conducted for 1 h at RT with agitation (200 rpm). Eluted phages were amplified in *E. coli* ER2738 cells and purified by PEG-8000/NaCl precipitation. The purified phages were used for the subsequent round of selection. Three rounds of selection were performed to enrich for high-affinity clones. Titration was carried out after each round to monitor the recovery rate and enrichment of phage clones specific to Activin A.

### 4.3. Phage DNA Sequencing

Phage DNA was extracted from 24 clones selected after the phage–ELISA binding assay (Supplementary Figure S1). Overnight cultures of *E. coli* ER2738 infected with the selected phages were grown in a deep-well plate. After centrifugation to pellet the bacterial cells, the supernatant was transferred to a fresh plate. Phages were precipitated by adding 1/6 (*v*/*v*) PEG-8000/NaCl solution, followed by centrifugation. The resulting phage pellet was resuspended in 100 µL of sodium iodide (NaI) buffer (10 mM Tris-HCl, pH 8.0; 1 mM EDTA; 4 M NaI) to promote lysis [70].

Lysates were precipitated with absolute ethanol for 10 min and centrifuged at 10,000 rpm, and the DNA pellet was washed with 70% ethanol. The DNA was resuspended in 15 µL of Milli-Q water and quality-checked using 0.8% agarose gel electrophoresis. DNA concentration was measured using a NanoDrop™ 1000 spectrophotometer (Thermo Fisher Scientific, Waltham, MA, USA). Sequencing was performed by the Myleus Biotechnology Facility (Belo Horizonte, Brazil) using a 3730 DNA Analyzer (Thermo Fisher Scientific, Waltham, MA, USA). For sequencing, 100 ng of phage DNA was mixed with 1 µL of M13 sequencing primer (10 µmol, 5′-OH CCC TCA TAG TTA GCG TAA CG-3′, New England Biolabs Inc., Ipswich, MA, USA) and Tris-EDTA buffer solution to a final volume of 7.5 µL, following the facility’s guidelines.

### 4.4. In Silico Analysis

DNA sequences obtained from phage DNA sequencing were translated into peptide sequences using the ExPASy Translate tool (https://web.expasy.org/translate/, accessed on 11 October 2016) [71]. The predicted amino acid sequences were then used to model the three-dimensional structures of the peptides. Structural predictions were performed using PepFold3 (https://bioserv.rpbs.univ-paris-diderot.fr/services/PEP-FOLD3/, accessed on 11 October 2016) [72,73,74], an established tool for de novo peptide structure prediction. The resulting 3D models were visualized and analyzed using PyMOL (https://pymol.org/, accessed on 18 October 2016), which provided insights into peptide conformations and spatial arrangements. To evaluate the structural interactions and docking between the selected peptides and the Activin A molecule, the online platform GalaxyPepDock (https://seoklab.org/GalaxyPepDock/, accessed on 7 November 2016) was utilized [75]. GalaxyPepDock was also used to assess interactions and docking predictions between Activin A and its natural inhibitor follistatin, as well as Activin A and its receptor ActRIIB. These docking analyses provided insights into potential binding regions, interaction energies, and key residues involved in the interactions. All tools used were publicly available, and the default parameters recommended by each software were applied unless otherwise specified. The analyses aimed to identify peptides with strong and specific binding to Activin A, particularly in regions critical for its interaction with follistatin and ActRIIB.

### 4.5. Peptide Synthesis

Following the results of the in vitro and in silico analyses, peptides A7, B9, and E10 were selected for synthesis. These peptides were synthesized by GenScript (Piscataway, NJ, USA) using solid-phase peptide synthesis (SPPS) technology. Each peptide was synthesized with >95% purity, as confirmed by high-performance liquid chromatography (HPLC), and the final structures were verified using mass spectrometry. Peptides were synthesized with N-terminal acetylation and C-terminal amidation to enhance stability and mimic natural peptide configurations, following the phage display manual [70]. Lyophilized peptides were provided in powder form and reconstituted in sterile phosphate-buffered saline (PBS) to achieve working concentrations.

### 4.6. Cell Culture Assays

For cell culture assays, A549 cells and primary fibroblasts were maintained in high-glucose Dulbecco’s Modified Eagle Medium (DMEM) (Gibco, Thermo Fisher Scientific, Waltham, MA, USA) supplemented with 10% fetal bovine serum (FBS) (Sigma-Aldrich, St. Louis, MO, USA) and 1% gentamicin (Sigma-Aldrich, St. Louis, MO, USA). Cultures were incubated under standard conditions at 37 °C in a humidified atmosphere containing 5% CO_2_. The culture medium was replaced every three days to ensure optimal nutrient availability and to remove cellular waste. Cells were passaged when they reached approximately 80% confluence using 0.05% trypsin-EDTA (Gibco, Thermo Fisher Scientific, Waltham, MA, USA) for detachment. Experiments were initiated when cells reached the required confluence, depending on the specific assay.

#### 4.6.1. Cytotoxicity Assay by MTT

A total of 1 × 10^4^ A549 cells were seeded into each well of a 96-well plate in 100 µL of high-glucose DMEM (Gibco, Thermo Fisher Scientific, Waltham, MA, USA) supplemented with 10% FBS (Sigma-Aldrich, St. Louis, MO, USA) and 1% gentamicin (Sigma-Aldrich, St. Louis, MO, USA) (complete medium). Cells were incubated under standard culture conditions (37 °C, 95% humidified air, and 5% CO_2_) for 24 h. After the initial incubation, the medium was replaced with serum-free high-glucose DMEM to synchronize the cell cycles. Cells were then treated with synthetic peptides (A7, B9, and E10) at concentrations of 1 μM, 10 μM, and 50 μM for 24, 48, and 72 h. Groups were also evaluated with the addition of pm26TGF-β1 (1 ng/mL), a peptide that mimics the effects of TGF-β1, to mimic a fibrotic stimulus [69].

At each time point, 10 μL of 3-(4,5-dimethylthiazol-2-yl)-2,5-diphenyltetrazolium bromide (MTT) solution (5 mg/mL, Calbiochem, Darmstadt, Germany) was added to each well, followed by incubation at 37 °C for 4 h. Formazan crystals were dissolved by adding 50 μL of N-dimethylmethanamide to each well, and the plate was incubated overnight. Absorbance was measured at 592 nm using a microplate reader (Titertek Multiskan Plus, Flow Laboratories, McLean, VA, USA).

The relative cell viability (%) was calculated using the following formula:

%*Viability* = (*A*592(*untreated cells*)/*A592*(*treated cells*)) × 100

Control groups were included as follows: negative control cells received serum-free DMEM only, while positive control cells were treated with pm26TGF-β1 (1 ng/mL) to mimic a fibrotic stimulus. All experiments were conducted in triplicate to ensure statistical reliability.

#### 4.6.2. Cellular Migration

A total of 1 × 10⁴ A549 cells were cultured in a 24-well plate in high-glucose DMEM (Gibco, Thermo Fisher Scientific, Waltham, MA, USA) supplemented with 10% FBS and 1% gentamicin (Sigma-Aldrich, St. Louis, MO, USA) under standard culture conditions (37 °C, 95% humidified air, and 5% CO_2_). Cells were grown to approximately 80% confluence before a scratch was made in the monolayer using a sterile P200 pipette tip. Detached cells and debris were removed by washing the wells twice with PBS 1X. Cells were then treated with high-glucose DMEM complete medium containing synthetic peptides A7, B9, or E10 at concentrations of 1 μM, 10 μM, and 50 μM. Groups were also evaluated with the addition of pm26TGF-β1 (1 ng/mL), a peptide that mimics the effects of TGF-β1, to mimic a fibrotic stimulus [69].

Images of the scratch area were captured at 0, 24, and 48 h using an EVOS^®^ FL Auto Imaging System (Thermo Fisher Scientific, Wilmington, DE, USA). Cellular migration was quantified by comparing the wound area at each time point to the initial scratch area using ImageJ software (v1.53k). Results are expressed as the percentage of wound closure, calculated as follows:

%*Wound Closure* = ((*Initial Area* − *Final Area*) / *Initial Area*) × 100 (1)

Control groups were included as follows: negative control cells received DMEM only, while positive control cells were treated with pm26TGF-β1 (1 ng/mL) without peptide treatment. All experiments were conducted in triplicate to ensure statistical reliability.

#### 4.6.3. Fibroblast-to-Myofibroblast Transition (FMT) Assay

Primary human fibroblasts (~2500 cells/well) were plated in 96-well plates and maintained under standard culture conditions (37 °C, 95% humidified air, and 5% CO_2_) in high-glucose DMEM (Gibco, Thermo Fisher Scientific, Waltham, MA, USA) supplemented with 10% FBS (Sigma-Aldrich, St. Louis, MO, USA). Media were changed every two days. On day four, the medium was replaced with serum-free high-glucose DMEM containing pm26TGF-β1 (1 ng/mL) to induce FMT, along with peptide E10 at 1 µM, 10 µM, or 50 µM. Cells were incubated for an additional 72 h under standard culture conditions. Negative control cells were cultured in serum-free DMEM without pm26TGF-β1 or peptide treatment, while positive control cells were treated with pm26TGF-β1 (1 ng/mL) alone.

After treatment, cells were fixed with 4% formaldehyde at room temperature for 15 min and washed three times with PBS 1X. Cells were permeabilized with 100% chilled ethanol for 10 min, followed by washing twice with PBS 1X. Blocking was performed with 5% PBS-BSA solution for 1 h at 4 °C. Cells were incubated overnight at 4 °C with anti-α-SMA primary antibody (ab7817, Abcam, Cambridge, MA, USA) and diluted according to the manufacturer’s instructions while protected from light. After incubation, cells were washed three times with PBS 1X and incubated with anti-mouse IgG-FITC secondary antibody (Merck, Darmstadt, Germany) for 1 h at 37 °C in the dark.

To visualize nuclei, cells were treated with 100 µL of DAPI (4′,6-Diamidino-2-phenylindole) solution (1 µg/mL) for 15 min, followed by an additional PBS 1X washing step. Images were captured using an EVOS^®^ FL Auto Imaging System (Thermo Fisher Scientific, Wilmington, DE, USA). The assay was performed in triplicate for each experimental condition.

#### 4.6.4. Epithelial-to-Mesenchymal Transition (EMT) Assay

A549 cells (~2500 cells/well) were plated in 96-well plates and maintained under standard culture conditions (37 °C, 95% humidified air, and 5% CO_2_) in high-glucose DMEM (Gibco, Thermo Fisher Scientific, Waltham, MA, USA) supplemented with 10% FBS (Sigma-Aldrich, St. Louis, MO, USA). Media were changed every two days. On day four, the medium was replaced with serum-free high-glucose DMEM supplemented with pm26TGF-β1 (1 ng/mL) to induce EMT, along with peptide E10 at 1 µM, 10 µM, or 50 µM. Cells were incubated for an additional 72 h under standard culture conditions. Negative control cells were cultured in serum-free DMEM without pm26TGF-β1 or peptide treatment, while positive control cells were treated with pm26TGF-β1 (1 ng/mL) alone.

After treatment, cells were fixed with 4% formaldehyde at room temperature for 15 min and washed three times with PBS 1X. Cells were permeabilized with chilled ethanol for 10 min, followed by two additional washes with PBS 1X. Blocking was performed using 5% PBS-BSA solution for 1 h at 4 °C. Cells were incubated overnight at 4 °C with anti-fibronectin primary antibody (ab6328, Abcam, Cambridge, MA, USA) and diluted according to the manufacturer’s instructions while protected from light.

After overnight incubation, cells were washed three times with PBS 1X and incubated with anti-mouse IgG Atto 647 secondary antibody (Merck, Darmstadt, Germany) for 1 h at 37 °C in the dark. Following this, cells were treated with 100 µL of DAPI solution (1 µg/mL) for 15 min to stain the nuclei. Excess DAPI was removed by an additional washing step with PBS 1X. Images were captured using an EVOS^®^ FL Auto Imaging System (Thermo Fisher Scientific, Wilmington, DE, USA). All experiments were conducted in triplicate for each experimental condition.

#### 4.6.5. Statistical Analyses

All statistical analyses were conducted using R (version 4.2.0) and relevant libraries, including ggplot2, dplyr, and rstatix. Data are presented as mean ± standard deviation (SD) from at least three independent experiments. Statistical comparisons were performed using appropriate tests based on the experimental design and data distribution.

For pairwise comparisons, unpaired Student’s *t*-tests were applied. In the cytotoxicity and cellular migration assays, the False Discovery Rate (FDR) method was applied for *p*-value adjustments to control for multiple comparisons. Statistical significance was defined as an adjusted *p*-value < 0.05, with results reported as * *p* < 0.05, ** *p* < 0.01, and *** *p* < 0.001. Data visualization was performed using the ggplot2 package v. 3.5.1. Detailed R scripts for analysis are available upon request to ensure transparency and reproducibility.

## 5. Conclusions

This study identifies and characterizes peptide E10 as a promising inhibitor of Activin A, a critical mediator in the pathogenesis of idiopathic pulmonary fibrosis (IPF). Through a combination of in vitro, in silico, and cell-based assays, E10 demonstrated high binding specificity to Activin A and effectively inhibited key fibrotic processes, including EMT, FMT, and cellular migration. Importantly, E10 exhibited minimal cytotoxicity, further supporting its potential for therapeutic application.

The findings demonstrate the utility of phage display in identifying high-affinity inhibitors and highlight E10’s potential as a therapeutic agent for IPF. By interfering with upstream drivers of fibrosis, E10 offers a novel approach compared to existing treatments, which primarily target downstream pathways with limited efficacy.

Future research should focus on validating the efficacy and safety of E10 in vivo, exploring its pharmacokinetics, and investigating its potential synergy with existing antifibrotic therapies. The development of peptide-based therapeutics like E10 could significantly advance the treatment landscape for IPF, offering hope for improved outcomes in a disease that currently has limited options.

In conclusion, this study lays a strong foundation for the continued exploration of peptide E10 as a targeted antifibrotic therapy, potentially paving the way for innovative approaches to combat IPF and other fibrotic diseases.

## 6. Patents

The findings of this study have been secured under patent application BR 10 2018 071971-8, titled “Peptídeos Ligantes e Inibidores de Activina A e Suas Aplicações Diagnósticas e Terapêuticas” (“Peptides Binding and Inhibiting Activin A and Their Diagnostic and Therapeutic Applications”), filed on 25 October 2018. This patent application was jointly submitted by the Federal University of Uberlândia (UFU) and the Research Support Foundation of Minas Gerais (FAPEMIG). It describes the identification, characterization, and potential therapeutic applications of peptides specifically designed to bind and inhibit Activin A, targeting its pathological role in IPF and other fibrotic diseases.

The invention employs phage display technology to identify highly specific peptides, highlighting three exemplary sequences for their strong affinity and specificity toward Activin A. The patent further proposes the use of these peptides in various diagnostic and therapeutic platforms, including ELISA-based assays, imaging modalities, and as targeted drug delivery agents. The described peptides have demonstrated potential for use in mitigating fibrotic progression by interfering with Activin A signaling pathways. Additionally, their application spans diverse diagnostic and therapeutic tools aimed at improving IPF management.

The protection offered by this patent underscores the innovation and translational potential of the research, paving the way for novel therapeutic strategies against IPF and related conditions.

## Data Availability

The original contributions presented in this study are included in this article/Supplementary Materials; further inquiries can be directed to the corresponding author.

## Supplementary Materials

The following supporting information can be downloaded at: https://www.mdpi.com/article/10.3390/ijms26062705/s1.

## References

[1] Urteaga M.B., Ruz J.R., González M.S. (2022). Idiopathic Pulmonary Fibrosis. Radiol. (Engl. Ed.).

[2] Spagnolo P., Kropski J.A., Jones M.G., Lee J.S., Rossi G., Karampitsakos T., Maher T.M., Tzouvelekis A., Ryerson C.J. (2021). Idiopathic Pulmonary Fibrosis: Disease Mechanisms and Drug Development. Pharmacol. Ther..

[3] George P.M., Spagnolo P., Kreuter M., Altinisik G., Bonifazi M., Martinez F.J., Molyneaux P.L., Renzoni E.A., Richeldi L., Tomassetti S. (2020). Progressive Fibrosing Interstitial Lung Disease: Clinical Uncertainties, Consensus Recommendations, and Research Priorities. Lancet Respir. Med..

[4] Koudstaal T., Wijsenbeek M.S. (2023). Idiopathic Pulmonary Fibrosis. Presse Medicale.

[5] Glass D.S., Grossfeld D., Renna H.A., Agarwala P., Spiegler P., DeLeon J., Reiss A.B. (2022). Idiopathic Pulmonary Fibrosis: Current and Future Treatment. Clin. Respir. J..

[6] Podolanczuk A.J., Thomson C.C., Remy-Jardin M., Richeldi L., Martinez F.J., Kolb M., Raghu G. (2022). Idiopathic Pulmonary Fibrosis: State of the Art for 2023. Eur. Respir. J..

[7] Santos G., Fabiano A., Mota P.C., Rodrigues I., Carvalho D., Melo N., Novais-Bastos H., Alexandre A.T., Moura C.S., Guimarães S. (2023). The Impact of Nintedanib and Pirfenidone on Lung Function and Survival in Patients with Idiopathic Pulmonary Fibrosis in Real-Life Setting. Pulm. Pharmacol. Ther..

[8] Liu J., Wang F., Hong Y., Luo F. (2024). Bibliometric Analysis of the Pirfenidone and Nintedanib in Interstitial Lung Diseases. Heliyon.

[9] Morianos I., Papadopoulou G., Semitekolou M., Xanthou G. (2019). Activin-A in the Regulation of Immunity in Health and Disease. J. Autoimmun..

[10] Pan T., Feng Y., Li Y., Yang Y., Zhou J., Song Y. (2024). Exacerbation of Pulmonary Fibrosis Following Acute Lung Injury via Activin-A Production by Recruited Alveolar Macrophages. J. Thorac. Dis..

[11] Karagiannidis C., Hense G., Martin C., Epstein M., Rückert B., Mantel P.-Y., Menz G., Uhlig S., Blaser K., Schmidt-Weber C.B. (2006). Activin A Is an Acute Allergen-Responsive Cytokine and Provides a Link to TGF-β–Mediated Airway Remodeling in Asthma. J. Allergy Clin. Immunol..

[12] Kundra S., Kaur R., Pasricha C., Kumari P., Singh T.G., Singh R. (2024). Pathological Insights into Activin A: Molecular Underpinnings and Therapeutic Prospects in Various Diseases. Int. Immunopharmacol..

[13] Jones K.L., de Kretser D.M., Patella S., Phillips D.J. (2004). Activin A and Follistatin in Systemic Inflammation. Mol. Cell. Endocrinol..

[14] Xie T., Han L., Chen Y., Wu H. (2021). Progranulin and Activin A Concentrations Are Elevated in Serum from Patients with Acute Exacerbations of Idiopathic Pulmonary Fibrosis. Lung.

[15] Zhang Z., Wang J., Chen Y., Suo L., Chen H., Zhu L., Wan G., Han X. (2019). Activin a Promotes Myofibroblast Differentiation of Endometrial Mesenchymal Stem Cells via STAT3-Dependent Smad/CTGF Pathway. Cell Commun. Signal..

[16] Younesi F.S., Miller A.E., Barker T.H., Rossi F.M.V., Hinz B. (2024). Fibroblast and Myofibroblast Activation in Normal Tissue Repair and Fibrosis. Nat. Rev. Mol. Cell Biol..

[17] Hardy C.L., King S.J., Mifsud N.A., Hedger M.P., Phillips D.J., Mackay F., de Kretser D.M., Wilson J.W., Rolland J.M., O’Hehir R.E. (2015). The Activin A Antagonist Follistatin Inhibits Cystic Fibrosis-like Lung Inflammation and Pathology. Immunol. Cell Biol..

[18] Wietecha M.S., Pensalfini M., Cangkrama M., Müller B., Jin J., Brinckmann J., Mazza E., Werner S. (2020). Activin-Mediated Alterations of the Fibroblast Transcriptome and Matrisome Control the Biomechanical Properties of Skin Wounds. Nat. Commun..

[19] Nagayama I., Takei Y., Takahashi S., Okada M., Maeshima A. (2024). The Activin-Follistatin System: Key Regulator of Kidney Development, Regeneration, Inflammation, and Fibrosis. Cytokine Growth Factor Rev..

[20] Harrington A.E., Morris-Triggs S.A., Ruotolo B.T., Robinson C.V., Ohnuma S., Hyvönen M. (2006). Structural Basis for the Inhibition of Activin Signalling by Follistatin. EMBO J..

[21] Lamper A.M., Fleming R.H., Ladd K.M., Lee A.S.Y. (2020). A Phosphorylation-Regulated EIF3d Translation Switch Mediates Cellular Adaptation to Metabolic Stress. Science.

[22] Rozans S.J., Moghaddam A.S., Wu Y., Atanasoff K., Nino L., Dunne K., Pashuck E.T. (2024). Quantifying and Controlling the Proteolytic Degradation of Cell Adhesion Peptides. ACS Biomater. Sci. Eng..

[23] Rozans S.J., Moghaddam A.S., Pashuck E.T. (2024). A Streamlined High-Throughput LC-MS Assay for Quantifying Peptide Degradation in Cell Culture. bioRxiv.

[24] Bonella F., Spagnolo P., Ryerson C. (2023). Current and Future Treatment Landscape for Idiopathic Pulmonary Fibrosis. Drugs.

[25] Guo H., Sun J., Zhang S., Nie Y., Zhou S., Zeng Y. (2023). Progress in Understanding and Treating Idiopathic Pulmonary Fibrosis: Recent Insights and Emerging Therapies. Front. Pharmacol..

[26] Chianese M., Screm G., Salton F., Confalonieri P., Trotta L., Barbieri M., Ruggero L., Mari M., Reccardini N., Geri P. (2024). Pirfenidone and Nintedanib in Pulmonary Fibrosis: Lights and Shadows. Pharmaceuticals.

[27] Kou M., Jiao Y., Li Z., Wei B., Li Y., Cai Y., Wei W. (2024). Real-World Safety and Effectiveness of Pirfenidone and Nintedanib in the Treatment of Idiopathic Pulmonary Fibrosis: A Systematic Review and Meta-Analysis. Eur. J. Clin. Pharmacol..

[28] Singh S., Wairkar S. (2024). Revolutionizing the Treatment of Idiopathic Pulmonary Fibrosis: From Conventional Therapies to Advanced Drug Delivery Systems. AAPS PharmSciTech.

[29] Molina-Molina M. (2019). The Future of Pharmacological Treatment in Idiopathic Pulmonary Fibrosis. Arch. Bronconeumol. (Engl. Ed.).

[30] Libra A., Sciacca E., Muscato G., Sambataro G., Spicuzza L., Vancheri C. (2024). Highlights on Future Treatments of IPF: Clues and Pitfalls. Int. J. Mol. Sci..

[31] Shakeel I., Afzal M., Islam A., Sohal S.S., Hassan M.I. (2023). Idiopathic Pulmonary Fibrosis: Pathophysiology, Cellular Signaling, Diagnostic and Therapeutic Approaches. Accel. Drug Discov..

[32] Namwanje M., Brown C.W. (2016). Activins and Inhibins: Roles in Development, Physiology, and Disease. CSH Perspect. Biol..

[33] Xia Y., Schneyer A.L. (2009). The Biology of Activin: Recent Advances in Structure, Regulation and Function. J. Endocrinol..

[34] Bao H., Sin T.K., Zhang G. (2018). Activin A Induces Leiomyoma Cell Proliferation, Extracellular Matrix (ECM) Accumulation and Myofibroblastic Transformation of Myometrial Cells via P38 MAPK. Biochem. Biophys. Res. Commun..

[35] Yuan C., Ni L., Wu X. (2021). Activin A Activation Drives Renal Fibrosis through the STAT3 Signaling Pathway. Int. J. Biochem. Cell Biol..

[36] Li S., Li Z., Yin R., Nie J., Fu Y., Ying R. (2021). Knockdown of Dual Oxidase 1 Suppresses Activin A-Induced Fibrosis in Cardiomyocytes via the Reactive Oxygen Species-Dependent Pyroptotic Pathway. Int. J. Biochem. Cell Biol..

[37] Ren L.-L., Li X.-J., Duan T.-T., Li Z.-H., Yang J.-Z., Zhang Y.-M., Zou L., Miao H., Zhao Y.-Y. (2023). Transforming Growth Factor-β Signaling: From Tissue Fibrosis to Therapeutic Opportunities. Chem. Biol. Interact..

[38] Aoki F., Kurabayashi M., Hasegawa Y., Kojima I. (2005). Attenuation of Bleomycin-Induced Pulmonary Fibrosis by Follistatin. Am. J. Respir. Crit. Care Med..

[39] Lee S.-J., Bhasin S., Klickstein L., Krishnan V., Rooks D. (2023). Challenges and Future Prospects of Targeting Myostatin/Activin A Signaling to Treat Diseases of Muscle Loss and Metabolic Dysfunction. J. Gerontol. Ser. A.

[40] Yaden B.C., Wang Y.X., Wilson J.M., Culver A.E., Milner A., Datta-Mannan A., Shetler P., Croy J.E., Dai G., Krishnan V. (2014). Inhibition of Activin A Ameliorates Skeletal Muscle Injury and Rescues Contractile Properties by Inducing Efficient Remodeling in Female Mice. Am. J. Pathol..

[41] Polkey M.I., Praestgaard J., Berwick A., Franssen F.M.E., Singh D., Steiner M.C., Casaburi R., Tillmann H.-C., Lach-Trifilieff E., Roubenoff R. (2019). Activin Type II Receptor Blockade for Treatment of Muscle Depletion in Chronic Obstructive Pulmonary Disease. A Randomized Trial. Am. J. Respir. Crit. Care Med..

[42] Verma A., Suragani R.N.V.S., Aluri S., Shah N., Bhagat T.D., Alexander M.J., Komrokji R., Kumar R. (2020). Biological Basis for Efficacy of Activin Receptor Ligand Traps in Myelodysplastic Syndromes. J. Clin. Investig..

[43] Li F., Long Y., Yu X., Tong Y., Gong L. (2022). Different Immunoregulation Roles of Activin A Compared With TGF-β. Front. Immunol..

[44] Rossino G., Marchese E., Galli G., Verde F., Finizio M., Serra M., Linciano P., Collina S. (2023). Peptides as Therapeutic Agents: Challenges and Opportunities in the Green Transition Era. Molecules.

[45] Cabri W., Cantelmi P., Corbisiero D., Fantoni T., Ferrazzano L., Martelli G., Mattellone A., Tolomelli A. (2021). Therapeutic Peptides Targeting PPI in Clinical Development: Overview, Mechanism of Action and Perspectives. Front. Mol. Biosci..

[46] Karande S., Sharma K., Kumar A., Charan S., Patil C., Sharma A. (2023). Potential Role of Biopeptides in the Treatment of Idiopathic Pulmonary Fibrosis. Health Sci. Rev..

[47] Mason A.J. (1994). Functional Analysis of the Cysteine Residues of Activin A. Mol. Endocrinol..

[48] Harrison C.A., Gray P.C., Vale W.W., Robertson D.M. (2005). Antagonists of Activin Signaling: Mechanisms and Potential Biological Applications. Trends Endocrinol. Metab..

[49] Thompson T.B., Woodruff T.K., Jardetzky T.S. (2003). Structures of an ActRIIB:Activin A Complex Reveal a Novel Binding Mode for TGF-β Ligand:Receptor Interactions. EMBO J..

[50] Fischer W., Park M., Donaldson C., Wiater E., Vaughan J., Bilezikjian L., Vale W. (2003). Residues in the C-Terminal Region of Activin A Determine Specificity for Follistatin and Type II Receptor Binding. J. Endocrinol..

[51] Ding H., Chen J., Qin J., Chen R., Yi Z. (2021). TGF-β-Induced α-SMA Expression Is Mediated by C/EBPβ Acetylation in Human Alveolar Epithelial Cells. Mol. Med..

[52] Pulivendala G., Bale S., Godugu C. (2020). Honokiol: A Polyphenol Neolignan Ameliorates Pulmonary Fibrosis by Inhibiting TGF-β/Smad Signaling, Matrix Proteins and IL-6/CD44/STAT3 Axis Both in vitro and in vivo. Toxicol. Appl. Pharmacol..

[53] Ding Y., Hou Y., Liu Y., Yu T., Cui Y., Nie H. (2022). MiR-130a-3p Alleviates Inflammatory and Fibrotic Phases of Pulmonary Fibrosis Through Proinflammatory Factor TNF-α and Profibrogenic Receptor TGF-ΒRII. Front. Pharmacol..

[54] Ruan H., Luan J., Gao S., Li S., Jiang Q., Liu R., Liang Q., Zhang R., Zhang F., Li X. (2021). Fedratinib Attenuates Bleomycin-Induced Pulmonary Fibrosis via the JAK2/STAT3 and TGF-Β1 Signaling Pathway. Molecules.

[55] Doerner A.M., Zuraw B.L. (2009). TGF-Β1 Induced Epithelial to Mesenchymal Transition (EMT) in Human Bronchial Epithelial Cells Is Enhanced by IL-1β but Not Abrogated by Corticosteroids. Respir. Res..

[56] Kasai H., Allen J.T., Mason R.M., Kamimura T., Zhang Z. (2005). TGF-Β1 Induces Human Alveolar Epithelial to Mesenchymal Cell Transition (EMT). Respir. Res..

[57] Sozzani S., Musso T. (2011). The Yin and Yang of Activin A. Blood.

[58] Frangogiannis N.G. (2016). Fibroblast—Extracellular Matrix Interactions in Tissue Fibrosis. Curr. Pathobiol. Rep..

[59] Li Y., Tang C.B., Kilian K.A. (2017). Matrix Mechanics Influence Fibroblast–Myofibroblast Transition by Directing the Localization of Histone Deacetylase 4. Cell. Mol. Bioeng..

[60] Horan G.S., Wood S., Ona V., Li D.J., Lukashev M.E., Weinreb P.H., Simon K.J., Hahm K., Allaire N.E., Rinaldi N.J. (2008). Partial Inhibition of Integrin Avβ6 Prevents Pulmonary Fibrosis without Exacerbating Inflammation. Am. J. Respir. Crit. Care Med..

[61] Xiong R., Geng B., Jiang W., Hu Y., Hu Z., Hao B., Li N., Geng Q. (2023). Histone Deacetylase 3 Deletion in Alveolar Type 2 Epithelial Cells Prevents Bleomycin-Induced Pulmonary Fibrosis. Clin. Epigenetics.

[62] Zhang C., Zhu X., Hua Y., Zhao Q., Wang K., Zhen L., Wang G., Lü J., Luo A., Cho W.C. (2019). YY1 Mediates TGF-Β1-Induced EMT and pro-Fibrogenesis in Alveolar Epithelial Cells. Respir. Res..

[63] Doolin M.T., Smith I.M., Stroka K.M. (2021). Fibroblast to Myofibroblast Transition Is Enhanced by Increased Cell Density. Mol. Biol. Cell.

[64] D’Urso M., Kurniawan N.A. (2020). Mechanical and Physical Regulation of Fibroblast–Myofibroblast Transition: From Cellular Mechanoresponse to Tissue Pathology. Front. Bioeng. Biotechnol..

[65] Gregorio J.D., Robuffo I., Spalletta S., Giambuzzi G., Iuliis V.D., Toniato E., Martinotti S., Conti P., Flati V. (2020). The Epithelial-to-Mesenchymal Transition as a Possible Therapeutic Target in Fibrotic Disorders. Front. Cell Dev. Biol..

[66] Kusama K., Fukushima Y., Yoshida K., Azumi M., Yoshie M., Mizuno Y., Kajihara T., Tamura K. (2021). PGE2 and Thrombin Induce Myofibroblast Transdifferentiation via Activin A and CTGF in Endometrial Stromal Cells. Endocrinology.

[67] Džepina P., Ćorić M., Perica M.K., Aničić M.N., Grizelj R., Vuković J. (2024). Expression of Activin A in Liver Tissue and the Outcome of Patients with Biliary Atresia. Front. Pediatr..

[68] He M., Yang T., Zhou J., Wang R., Li X. (2024). A Real-World Study of Antifibrotic Drugs-Related Adverse Events Based on the United States Food and Drug Administration Adverse Event Reporting System and VigiAccess Databases. Front. Pharmacol..

[69] Vaz E.R., Fujimura P.T., Araujo G.R., da Silva C.A.T., Silva R.L., Cunha T.M., Lopes-Ferreira M., Lima C., Ferreira M.J., Cunha-Junior J.P. (2015). A Short Peptide That Mimics the Binding Domain of TGF-Β1 Presents Potent Anti-Inflammatory Activity. PLoS ONE.

[70] Barbas C.F., Burton D.R., Scott J.K., Silverman G.J. (2001). Phage Display: A Laboratory Manua. Q. Rev. Biol..

[71] Gasteiger E., Gattiker A., Hoogland C., Ivanyi I., Appel R.D., Bairoch A. (2003). ExPASy: The Proteomics Server for in-Depth Protein Knowledge and Analysis. Nucleic Acids Res..

[72] Shen Y., Maupetit J., Derreumaux P., Tufféry P. (2014). Improved PEP-FOLD Approach for Peptide and Miniprotein Structure Prediction. J. Chem. Theory Comput..

[73] Thévenet P., Shen Y., Maupetit J., Guyon F., Derreumaux P., Tufféry P. (2012). PEP-FOLD: An Updated de Novo Structure Prediction Server for Both Linear and Disulfide Bonded Cyclic Peptides. Nucleic Acids Res..

[74] Lamiable A., Thévenet P., Rey J., Vavrusa M., Derreumaux P., Tufféry P. (2016). PEP-FOLD3: Faster de Novo Structure Prediction for Linear Peptides in Solution and in Complex. Nucleic Acids Res..

[75] Lee H., Heo L., Lee M.S., Seok C. (2015). GalaxyPepDock: A Protein–Peptide Docking Tool Based on Interaction Similarity and Energy Optimization. Nucleic Acids Res..

