# Supplementary material for: Activin A Inhibitory Peptides Suppress Fibrotic Pathways by Targeting Epithelial–Mesenchymal Transition and Fibroblast–Myofibroblast Transformation in Idiopathic Pulmonary Fibrosis
Source: Int J Mol Sci. 2025 Mar 17;26(6):2705. doi: 10.3390/ijms26062705 (PMC11942258; doi:10.3390/ijms26062705)
Supplement: Supplementary file 1 [file ijms-26-02705-s001.zip › ijms-3466649-supplementary.pdf]

International Journal of *Molecular Sciences*

MDPI

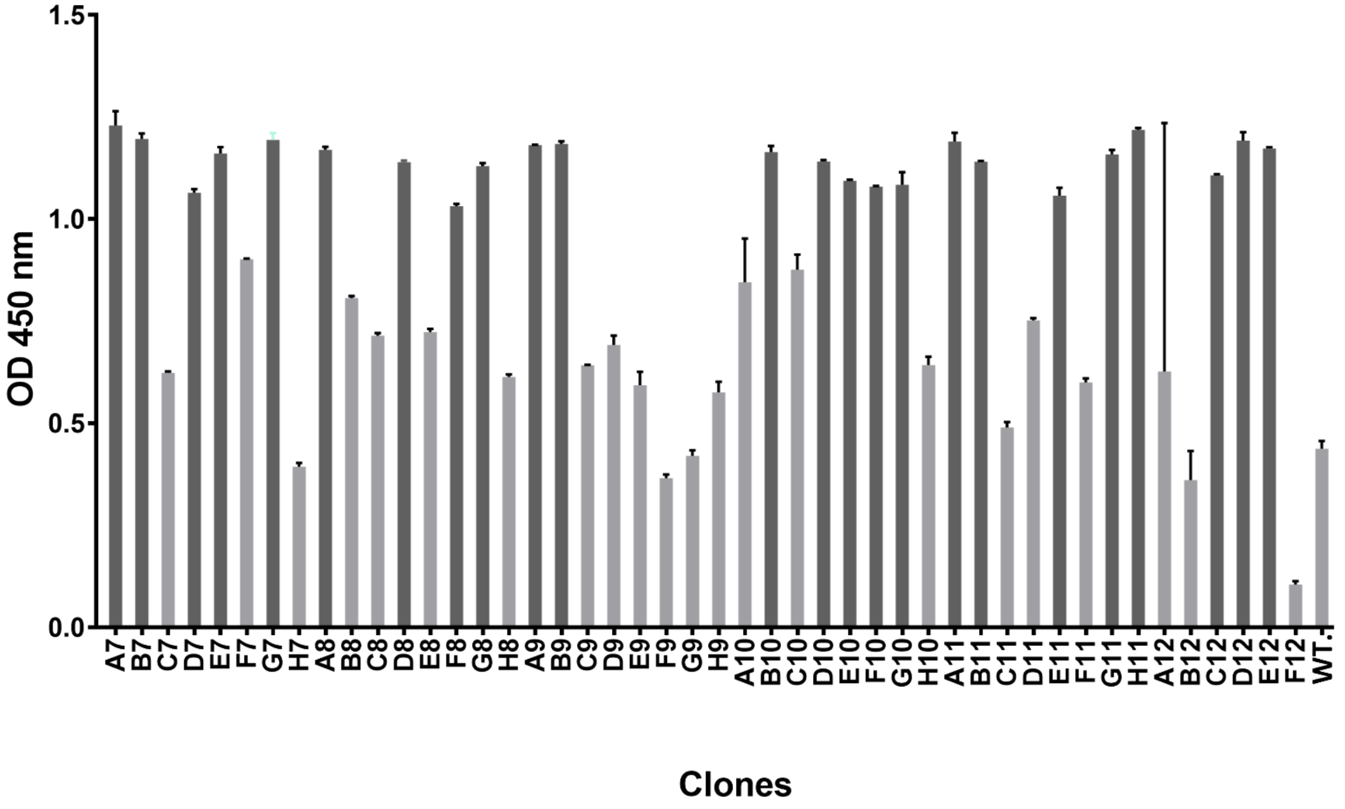

Figure S1: Evaluation in Phage-ELISA of the reactivity of the 46 selected clones using activin A as the target. Darker columns highlight clones with an ELISA index greater than two. 23 clones were selected for phage-DNA sequencing.

Table S1: Sequences obtained after phage-DNA sequencing.

Sequence	ID	Frequency (%)
PTPLAWL	A7	10 (43.48)
PGSLYWL	A8	2 (8.70)
PSTLLWL	D8	2 (8.70)
PDTLTWL	G10	2 (8.70)
PDSLRWL	D12	2 (8.70)
TFSVTNK	F8	1 (4.35)
PRELAFL	G8	1 (4.35)
PRDLLWL	B9	1 (4.35)
PPTLHWL	E10	1 (4.35)
PPSLKWI	F10	1 (4.35)

Table S2: Cytotoxicity assay data.

Peptide	Concentration (uM)	Condition	Mean Relative Viability	SE Relative Viability	p	p.signif	p.adj	p.adj.signif	Time
7	1	+ pmTGF-B	87.38493	6.7672777	0.1060000	ns	0.6290000	ns	24h
A7	1	- pmTGF-B	101.83611	4.5936744	0.7510000	ns	0.7510000	ns	24h
A7	10	+ pmTGF-B	91.74906	3.3765887	0.2940000	ns	0.7130000	ns	24h
A7	10	- pmTGF-B	95.44373	1.4052348	0.1020000	ns	0.1220000	ns	24h
A7	50	+ pmTGF-B	93.45380	1.0716832	0.0763000	ns	0.1140000	ns	24h
A7	50	- pmTGF-B	82.79497	4.0122407	0.0511000	ns	0.3070000	ns	24h
B9	1	+ pmTGF-B	90.86260	6.0489181	0.2240000	ns	0.6290000	ns	24h
B9	1	- pmTGF-B	105.64434	1.4052348	0.3410000	ns	0.5120000	ns	24h
B9	10	+ pmTGF-B	95.02216	6.3298873	0.5170000	ns	0.7130000	ns	24h
B9	10	- pmTGF-B	105.57633	0.5566374	0.0538000	ns	0.0806000	ns	24h
B9	50	+ pmTGF-B	88.68053	1.0044950	0.0078700	**	0.0236000	*	24h
B9	50	- pmTGF-B	89.79939	4.6357639	0.2110000	ns	0.4230000	ns	24h
Control	1	+ pmTGF-B	100.00000	3.5849818					24h
Control	1	- pmTGF-B	100.00000	1.9390109					24h
Control	10	+ pmTGF-B	100.00000	3.5849818					24h
Control	10	- pmTGF-B	100.00000	1.9390109					24h
Control	50	+ pmTGF-B	100.00000	3.5849818					24h
Control	50	- pmTGF-B	100.00000	1.9390109					24h
E10	1	+ pmTGF-B	92.56734	0.8705861	0.3150000	ns	0.6290000	ns	24h
E10	1	- pmTGF-B	92.99558	5.9506485	0.2450000	ns	0.4890000	ns	24h
E10	10	+ pmTGF-B	88.95329	6.6017221	0.1710000	ns	0.7130000	ns	24h
E10	10	- pmTGF-B	107.07242	2.4772534	0.0210000	*	0.0419000	*	24h
E10	50	+ pmTGF-B	86.08933	2.3866348	0.0025300	**	0.0152000	*	24h
E10	50	- pmTGF-B	94.08365	8.4470266	0.4530000	ns	0.5440000	ns	24h
A7	1	+ pmTGF-B	91.99445	6.7901182	0.2160000	ns	0.3130000	ns	48h
A7	1	- pmTGF-B	113.33333	4.6390192	0.2350000	ns	0.3520000	ns	48h
A7	10	+ pmTGF-B	80.93475	3.4551561	0.0061400	**	0.0151000	*	48h
A7	10	- pmTGF-B	100.00000	2.1837422	1.0000000	ns	1.0000000	ns	48h
A7	50	+ pmTGF-B	74.64137	2.2226395	0.0001630	***	0.0003260	***	48h
A7	50	- pmTGF-B	69.78142	3.0858597	0.0283000	*	0.1440000	ns	48h
B9	1	+ pmTGF-B	92.54975	4.6893140	0.2470000	ns	0.3130000	ns	48h
B9	1	- pmTGF-B	127.04918	5.1270111	0.0313000	*	0.1880000	ns	48h
B9	10	+ pmTGF-B	81.67515	2.5752289	0.0075600	**	0.0151000	*	48h
B9	10	- pmTGF-B	108.74317	5.7543345	0.3790000	ns	0.5690000	ns	48h
B9	50	+ pmTGF-B	64.73855	2.3481933	0.0000154	****	0.0000604	****	48h
B9	50	- pmTGF-B	73.60656	9.8170733	0.0479000	*	0.1440000	ns	48h
Control	1	+ pmTGF-B	100.00000	0.4897272					48h
Control	1	- pmTGF-B	100.00000	11.0333810					48h
Control	10	+ pmTGF-B	100.00000	0.4897272					48h
Control	10	- pmTGF-B	100.00000	11.0333810					48h
Control	50	+ pmTGF-B	100.00000	0.4897272					48h
Control	50	- pmTGF-B	100.00000	11.0333810					48h
E10	1	+ pmTGF-B	84.77557	1.6658954	0.0340000	*	0.2040000	ns	48h
E10	1	- pmTGF-B	118.19672	6.7620605	0.1180000	ns	0.3520000	ns	48h
E10	10	+ pmTGF-B	78.34336	5.8845556	0.0030400	**	0.0151000	*	48h
E10	10	- pmTGF-B	110.65574	4.1063674	0.2890000	ns	0.5690000	ns	48h
E10	50	+ pmTGF-B	65.98797	4.3030656	0.0000201	****	0.0000604	****	48h
E10	50	- pmTGF-B	79.72678	5.3081462	0.1110000	ns	0.2220000	ns	48h
A7	1	+ pmTGF-B	133.13180	3.1231553	0.0002200	***	0.0013200	**	72h
A7	1	- pmTGF-B	108.33062	2.5365669	0.2380000	ns	0.4760000	ns	72h

Peptide	Concentration (uM)	Condition	Mean Relative Viability	SE Relative Viability	p	p.signif	p.adj	p.adj.signif	Time
A7	10	+ pmTGF-B	111.76945	10.9692470	0.2540000	ns	0.8390000	ns	72h
A7	10	- pmTGF-B	89.26131	0.4064431	0.0432000	*	0.0865000	ns	72h
A7	50	+ pmTGF-B	71.86618	2.8852124	0.0031500	**	0.0094600	**	72h
A7	50	- pmTGF-B	70.64758	6.1869923	0.0394000	*	0.1500000	ns	72h
B9	1	+ pmTGF-B	119.99194	4.4395550	0.0049900	**	0.0099800	**	72h
B9	1	- pmTGF-B	101.69216	5.1043483	0.8020000	ns	0.8020000	ns	72h
B9	10	+ pmTGF-B	104.35308	3.0632809	0.6620000	ns	0.8390000	ns	72h
B9	10	- pmTGF-B	81.25610	1.3852129	0.0030500	**	0.0183000	*	72h
B9	50	+ pmTGF-B	62.67634	1.2410161	0.0005590	***	0.0033500	**	72h
B9	50	- pmTGF-B	72.46990	12.7301712	0.0500000	ns	0.1500000	ns	72h
Control	1	+ pmTGF-B	100.00000	4.8293642					72h
Control	1	- pmTGF-B	100.00000	1.7021408					72h
Control	10	+ pmTGF-B	100.00000	4.8293642					72h
Control	10	- pmTGF-B	100.00000	1.7021408					72h
Control	50	+ pmTGF-B	100.00000	4.8293642					72h
Control	50	- pmTGF-B	100.00000	1.7021408					72h
E10	1	+ pmTGF-B	113.13986	1.2592099	0.0358000	*	0.0430000	*	72h
E10	1	- pmTGF-B	110.73869	7.0731158	0.1390000	ns	0.4760000	ns	72h
E10	10	+ pmTGF-B	102.01532	5.5259373	0.8390000	ns	0.8390000	ns	72h
E10	10	- pmTGF-B	97.33160	5.9236240	0.5680000	ns	0.5680000	ns	72h
E10	50	+ pmTGF-B	84.36114	7.6275932	0.0494000	*	0.0741000	ns	72h
E10	50	- pmTGF-B	80.93069	9.0528531	0.1490000	ns	0.2980000	ns	72h
